# Role of ATP in migraine mechanisms: focus on P2X3 receptors

**DOI:** 10.1186/s10194-022-01535-4

**Published:** 2023-01-03

**Authors:** R. Giniatullin, A. Nistri

**Affiliations:** 1 A.I Virtanen Institute, University of Eastern Finland, 70211 Kuopio, Finland; 2 Department of Neuroscience, International School for Advanced Studies (SISSA), 34136 Trieste, Italy

**Keywords:** Migraine, Pain, Headache, ATP, P2X3, Trigeminal neurons, CGRP

## Abstract

Migraine is a major health burden worldwide with complex pathophysiology and multifarious underlying mechanisms. One poorly understood issue concerns the early steps in the generation of migraine pain. To elucidate the basic process of migraine pain further, it seems useful to consider key molecular players that may operate synergistically to evoke headache. While the neuropeptide CGRP is an important contributor, we propose that extracellular ATP (that generally plays a powerful nociceptive role) is also a major component of migraine headache, acting in concert with CGRP to stimulate trigeminal nociceptive neurons. The aim of the present focused review is to highlight the role of ATP activating its P2X3 membrane receptors selectively expressed by sensory neurons including their nerve fiber terminals in the meninges. Specifically, we present data on the homeostasis of ATP and related purines in the trigeminovascular system and in the CNS; the basic properties of ATP signalling at peripheral and central nerve terminals; the characteristics of P2X3 and related receptors in trigeminal neurons; the critical speed and persistence of P2X3 receptor activity; their cohabitation at the so-called meningeal neuro-immune synapse; the identity of certain endogenous agents cooperating with ATP to induce neuronal sensitization in the trigeminal sensory system; the role of P2X3 receptors in familial type migraine; the current state of P2X3 receptor antagonists and their pharmacological perspectives in migraine. It is proposed that the unique kinetic properties of P2X3 receptors activated by ATP offer an interesting translational value to stimulate future studies for innovative treatments of migraine pain.

## Background

The pathogenesis of migraine is complex since it involves interaction between peripheral and central neuronal mechanisms as highlighted in recent reviews [1–3]. One unresolved issue is the origin (and mechanism) of the typical pulsatile migraine pain, which is likely based on the activation of the meningeal trigeminovascular system [4–6]. To generate nociceptive signalling, which is further transmitted to the spinal cord/brainstem and to the higher pain centers, the trigeminal nerve terminals in the meninges should be first depolarized to a threshold sufficient to generate spiking activity [7]. To date, a lot of depolarizing stimuli were proposed to trigger such a depolarization [8] including extracellular ATP, serotonin, endovanilloids, low extracellular pH, mechanical forces and/or changes in the ambient temperature [7, 9, 10]. In addition to produce nociceptive firing, depolarization of meningeal peptidergic C-fibers can release calcitonin gene-related peptide (CGRP), which nowadays is considered a principal contributor to migraine attacks and an important target for migraine treatment [11–13]. The mode of action of CGRP is multifarious because it comprises activation of immune cells, control of meningeal vessels and facilitation of trigeminal afferent activity [6, 12, 14]. In particular, one key mechanism of CGRP action is sensitization of nociceptive trigeminal ganglion neurons that become hyper-responsive to various stimuli [15]. A major component of this phenomenon could be a strong upregulation of ATP-gated P2X3 receptors of trigeminal sensory neurons [16] and is one issue discussed in the present review.

While intracellular ATP is a purine compound essential for cell energy metabolism, extracellular ATP plays the role of neuromodulator/transmitter and is a potent pain-inducing agent [17–19]. Extracellular ATP acts on different subtypes of widely expressed ionotropic P2X and metabotropic P2Y receptors [20]. Among them, P2X2 and P2X3 subtypes expressed in sensory neurons can mediate local depolarization of nerve terminals and initiate propagating nociceptive signalling [21–23]. The purinergic hypothesis of migraine originally suggested by Burnstock had considered a vascular target for the ATP action [24, 25]. Later studies have shown that ATP can directly activate meningeal afferents [26–28] supporting the role of an ATP neuronal mechanism in migraine headache. Furthermore, because ATP is also released by glial cells and by neurons alone [29, 30] or together with other transmitters [31], its activity may be extended to key regions of the CNS implicated in migraine.

The current focused review, drawn from our research carried out with in vitro preparations of trigeminal ganglia and meningeal tissue, discusses the trigeminal sensory mechanisms likely underlying the algesic action of ATP and the potential role of P2X3 receptors (widely expressed by such neurons [32]) in migraine pathophysiology. Thus, the present data should be considered euristically to stimulate further research in vivo on this subject and any translational value to the clinic.

### Synthesis, release and degradation of ATP in migraine relevant tissues

Intracellular concentration of ATP is in the range of mM [33], while even higher levels of ATP can be found in synaptic vesicles as ATP is the co-transmitter released together with principal transmitters such as glutamate, noradrenaline, acetylcholine and GABA [31].

Apart from neuronal vesicular release, ATP can also be released from immune, vascular and glial cells or neurons through pannexin channels activated by mechanical forces or activation of specific receptors [34–36]. Pannexin-1 channels are functionally coupled with ATP-gated P2X7 receptors in the trigeminal ganglion [34]. Enhanced ATP release can also occur due to mechanical stimuli mediated by mechanosensitive Piezo channels expressed by neuronal and non-neuronal cells [37]. Indeed, it has recently been shown that Piezo1 channels of endothelial cells can provide flow-induced ATP release [38]. Moreover, Piezo1 channels are expressed in trigeminal neurons [39, 40] and it has been hypothesized they react to pulsatile blood flow by triggering spiking activity during a migraine attack [41]. It is tempting to speculate that direct mechanical activation of Piezo1 channels by pulsating vessels and ATP-dependent depolarization of meningeal afferents represent the basic mechanism of pulsatile migraine pain [39–42].

**Fig. 1:**
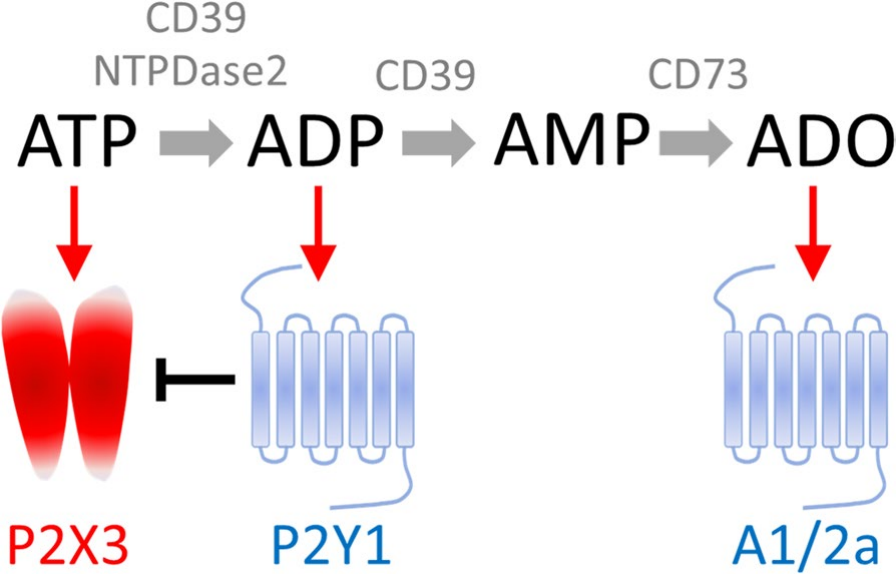
ATP degradation and migraine relevant signalling via P2X3, ADP-specific P2Y1 and adenosine activated A1 and A2a receptors. Extracellular ATP is degraded by CD39 to ADP and AMP and the latter is transformed to adenosine (ADO) by CD73. In contrast to CD39, neuron-specific NTPDase2 generates ADP. ATP can activate neuronal P2X3 receptors selectively expressed in sensory neurons, whereas ADP activates metabotropic neuronal P2Y1 receptors (probably also the P2Y12/13 subtypes), which in turn depress P2X3 receptor activity, thus completing this regulatory loop. Adenosine activates either inhibitory A1 or excitatory A2a receptors. Red arrows indicate activation while the block stop line shows an inhibitory effect

Extracellular ATP is very unstable and can provide only a short-lasting action as it is quickly broken down in living tissues by ectoenzymes [33] (Fig. 1). In addition to ATP breakdown to AMP by ecto-nucleoside triphosphate diphosphohydrolase-1 (NTPDase1/CD39), there are also other recently emerged extracellular enzymes (NTPDases2,3,8) including ATP-diphosphohydrolase, which can dephosphorylate ATP to the P2Y agonist ADP and the latter to the almost inactive AMP. The subsequent important step in this cascade is the degradation of AMP to physiologically active adenosine (ADO) by ecto-5'-nucleotidase/CD73 [33] (Fig. 1).

Production of ADP at the first step of ATP hydrolysis can activate ADP-preferring metabotropic P2Y1, P2Y12 and P2Y13 receptors, which are expressed in trigeminal neurons and in glial cells and can modulate nociception [43]. Interestingly, ADP can also provide an inhibitory effect on pro-nociceptive P2X3 receptors in sensory neurons [44] **(**Fig. 1). Unlike ATP, ADP does not excite meningeal afferents [26] and likely serves as negative feedback for ATP driven trigeminal nociception triggered by ionotropic receptors. This mechanism would originate from the local expression of ADP produced by NTPDases2,3,8 rather than by NTPDase1 mediated transformation of ATP to adenosine.

Adenosine can play either an anti-nociceptive role, via inhibitory A1 receptors widely expressed in neurons, or a pain triggering effect via activation of the cAMP-coupled A2a receptor subtype (Fig. 1). The latter operates via cAMP signalling to sensitize trigeminal neurons [45–47]. The modulatory action of ATP breakdown metabolites (ADP or adenosine) is expected to be most efficient because of the colocalization of specific NTPDases with the key components of the meningeal trigeminal nociceptive system such as vessels and nerves fibers. Our recently proposed approach, based on the detection of extracellular phosphate after ATP hydrolysis [26], has revealed ‘hot spots’ of intense ATP release/degradation around meningeal vessels surrounded by perivascular nerves.

Thus, the localization, subtype and activity of ATP degrading enzymes, the presence of downstream extracellular ATP metabolites and expression of specific receptors should all shape the functional outcome of purinergic pain signalling in migraine. This area of research is in progress and needs further studies.

### Basic properties of P2X3 receptors and their function in migraine mechanisms

The P2X3 receptor is the major ATP sensitive receptor subtype expressed in rodent trigeminal neurons (up to 80% of the whole population of trigeminal ganglion neurons in primary culture) [32]. Concerning trigeminal ganglion neurons innervating the rat dura mater, retrograde labelling revealed P2X3 or P2X2 subtype (or both) expressed in 52% neurons [48]. Extracellular ATP can operate at relatively low concentrations for activation of P2X3 receptors to which it has high affinity (EC_50_ ~ 1 μM) [49]. When extracellular ATP is not efficiently hydrolyzed, it can inhibit P2X3 receptor at low nanomolar concentrations through a mechanism known as the ‘high affinity desensitization’ (HAD) [50, 51]. HAD is a P2X3 specific phenomenon as it is not observed with the P2X2 receptor subtype [51]. Heteromerization of P2X3 subunits with slowly desensitizing P2X2 receptors is a common phenomenon in different types of sensory neurons [52, 53] and provides an additional transducer of nociceptive signals with lower adaptation.

The nerve fibers innervating meninges are nociceptive C- and Aδ-fibers [54–56] with their own repertoire of calcium, potassium and sodium channels plus nociceptive sensor proteins like P2X3 receptors [9, 26]. P2X3 receptors activated by ATP are highly expressed in nociceptive Aδ-fibers but also present in unmyelinated C-fibers [57, 58]. Indeed, in vivo topical application of ATP to rat meninges induces activation of approximately half population of C- and Aδ-fibers [28]. In the isolated rat hemiskull preparation, both ATP and the stable ATP analogue α,β-meATP (agonist of P2X1 and P2X3 receptor subtypes) induce sustained spiking activity in meningeal afferents [26, 59]. An even stronger effect of ATP is observed in mouse meningeal afferents [27]. Studies with the P2X2/3 antagonist A-317491 suggest that ATP may excite meningeal afferents via P2X3 and/or P2X2/3 receptors [26].

These data on the role of P2X2 and P2X3 receptors were obtained in in vitro conditions, when a prolonged application of exogenous ATP (or its analogues) only partially mimics the action of endogenous ATP which naturally takes place in restricted areas and is limited by the high activity of NTPDases. To overcome this experimental limitation, our modelling study [60] has simulated the action of endogenous ATP released from meningeal mast cells and has indicated that a sustained pro-nociceptive effect of ATP could be achieved via: *i*) multiple ATP release sites; *ii*) highly branched axon fibers; *iii*) coupling of desensitizing P2X3 receptors with slowly desensitizing P2X2 receptors; and *iv*) co-expression of Nav1.8 sodium channels that have fast recovery from voltage-dependent inactivation. While P2X2 receptors are less expressed in sensory neurons [32], especially in human ones [61], human P2X3 receptors recover from desensitization much faster than rodent ones [50], thus supporting a more persistent process for pain signalling.

In accordance with the International Classification of Headache Disorders (third edition), tension headache is another primary headache associated with tenderness of pericranial muscles [62]. Interestingly, injection of ATP into the trapezius muscle of a small group of healthy volunteers produces more pain compared to placebo [63]. Moreover, local injection of ATP (or a,b-meATP) into neck muscles induces strong, prolonged facilitation of nociceptive signaling in brainstem networks [64–66]. This effect of ATP is intensified after inhibition of ADP sensitive P2Y1 receptors [64] consistent with inhibitory control of P2X3 receptors by the ADP sensitive P2Y1 subtype [44] (Fig. 1). One possible mechanism of headache originating from neck muscles may be related to the branching of trigeminal neurons that can functionally connect intra- and extracranial areas [67, 68].

Branching of meningeal afferents could also contribute to enhanced antidromic sensory spiking by supplying signalling from axon collaterals or the trigeminal ganglion itself [69–71]. Antidromic spiking is supposed to initiate local CGRP release, vasodilation, and degranulation of mast cells, all events which are leading to sterile meningeal neuroinflammation [69]. Our recent study has provided direct evidence that spiking activity can actually be propagated from central trigeminal fibers to the peripheral terminals in the meninges [70]. Importantly, this study has shown that ATP receptors are present not only at the peripheral nerve terminals but also in more central parts of the nerve fibers extending our view on the principal mechanisms of peripheral nociception. We cannot exclude that P2X3 or P2X2/3 receptors are also widely expressed along the nonmyelinated C- fibers or located in the nodes of Ranvier of Aδ-fibers analogous to recently proposed location of CGRP receptors [72]. Taken together, these data cast some light on the involvement of P2X3 and P2X2/3 complexes in purinergic mechanisms contributing to trigeminal pain.

In the trigeminovascular system, in addition to nerve fibres, the local vessels and the process of nucleotide homeostasis (and signalling) may also be essential contributors to migraine pathology. These meningeal vessels, like other tissues constituting the trigeminovascular system, can be both a source and the target for the modulatory action of ATP. On such vessels, ATP can regulate the vascular tone directly via metabotropic P2Y13 and ionotropic P2X1 receptors promoting vasoconstriction [47, 73]. Conversely, the vessel vasodilatory effect of ATP may be observed after activation of P2X3 receptors on trigeminal neurons in the ganglion to trigger antidromic CGRP release in the meninges [47, 73]. Which one of the two contrasting actions is more relevant for migraine pain remains to be established.

In approximately 30% migraineurs the headache attack is preceded by an aura [74], i.e. a set of symptoms comprising visual dysfunction generated by a large wave of depolarization of the cerebral cortex termed cortical spreading depression (CSD) [5, 75]. A former study [76] has demonstrated that CSD is a sufficient trigger for ATP release in the cerebral cortex. The question then arises as to whether CSD might be a strong stimulus to release ATP at meningeal level as well. A direct answer to this question is currently missing. Nonetheless, it has been shown [28] that, in the fully anaesthetized rat, experimentally evoked CSD can activate about half of trigeminal nociceptors, a value similar to the responses to focally applied ATP. Even though this coincidence does not imply a causal link between two observations, these results suggest that ATP can induce excitation of trigeminal nociceptors in vivo even under deep anaesthesia. The implication of these data is that ATP-mediated activity might be important to understand different mechanisms of migraine with as well as without aura.

### Neuro-immune synapses and role of immune cells in meningeal nociception

**Fig. 2:**
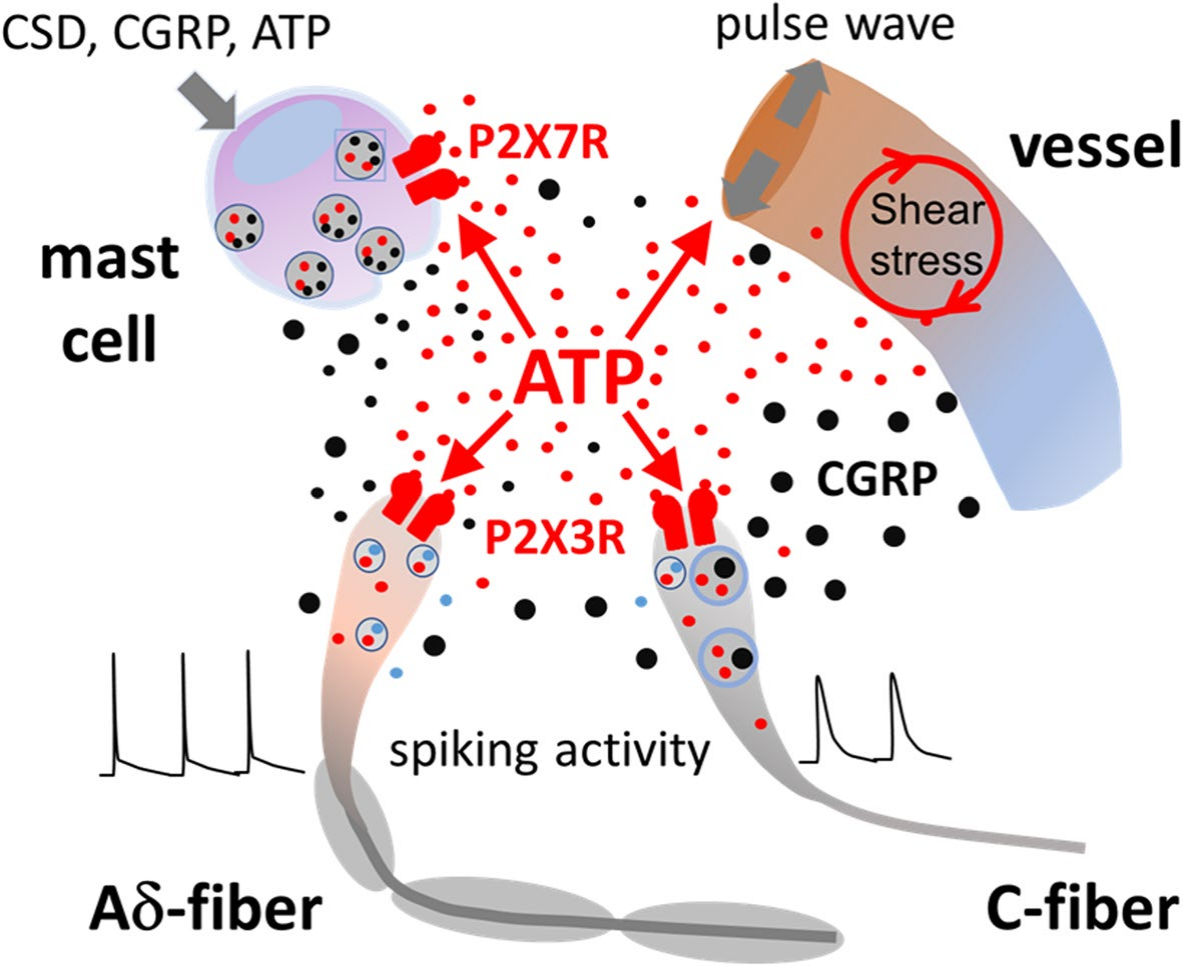
Proposed ATP-mediated pro-nociceptive signalling in the meninges involving trigeminal neurons, immune cells and local vessels. Meninges are densely innervated, colonized by local immune cells and largely vascularized. Extracellular ATP (small red circles) can: *i*) activate ionotropic P2X3 receptors in C- and Aδ-fibers [26, 86]; *ii*) degranulate mast cells via P2X7 receptors [27, 84]; *iii*) modulate vascular tone via metabotropic P2Y13 and P2X1 receptors [47, 73]. Other non-purinergic transmitters in this system, for sake of simplicity, are indicated only schematically as small black circles (for instance serotonin from mast cells) or small blue circles (glutamate from neurons)

Meningeal tissues contain various immune cells [77, 78]. Of special interest are local mast cells which synthesize a plethora of active molecules including ATP, hormones, cytokines, neurotrophins, all to be released in a stimulus-specific manner [79, 80]. These cells are located in close proximity to nerve fibers, forming a sort of meningeal neuro-immune synapse [81, 82] (Fig. 2). Notably, these mast cells could serve both as the target for ATP acting via P2X7 receptors [27, 83, 84] and as an additional source of ATP release [85].

The action of ATP on mast cells induces release of endogenous serotonin which can further amplify nociceptive signalling. Thus, ATP can combine two modes of nociceptive action on the meninges, namely, one through degranulating mast cells and release of serotonin to excite nerve terminals via 5-HT3 receptors, and the other one through a direct action on fibers via P2X3 receptors [27] (Fig. 2).

Immune cells in the meninges are associated with the local meningeal lymphatic system [87, 88] that provides a mechanism to clear metabolic waste from the brain and meninges themselves [89]. Consistent with the view that ATP, acting via P2X7 receptors, can trigger pro-inflammatory processes activating dural immune cells [27, 83, 84], we have observed that the P2X7-preferring agonist BzATP enhances release of the pro-inflammatory tumor necrosis factor-α (TNFα) and of the anti-inflammatory cytokine Il-10, both implicated in migraine pain [90, 91]. Nevertheless, the release of these cytokines is similar in WT and KO mice lacking the meningeal lymphatic system [92]. Furthermore, activation by ATP of meningeal afferents is not more effective in mice lacking meningeal lymphatics [92].

### P2X3 receptors and neuronal sensitization in migraine

While the neuropeptide CGRP is considered a major contributor to the onset of migraine attacks [12] and has multifarious effects on neurons and immune cells in the CNS [78], we propose that one important target for the CGRP algogenic action is the P2X3 receptor. Progress in understanding the molecular mechanisms of such an action of CGRP has come from the use of an in vitro model of mouse trigeminal ganglia where it has been shown that CGRP (at submicromolar concentrations) selectively binds to sensory neurons expressing P2X3 receptors without generating any direct change in their membrane current [16, 93]. Nonetheless, in the presence of CGRP, P2X3 receptor mediated responses become gradually larger with a significant upward shift of their agonist concentration response curve, indicating enhanced efficacy of P2X3 receptor activation [16]. It is important to note that this effect of CGRP has a delayed onset that develops over at least one hour and, therefore, mimics the slow, insidious onset of migraine headache when the peak concentration of serum CGRP in humans occurs about one hour after the start of pain [94]. The P2X3 receptor sensitization is obtained through a variety of mechanisms that comprise accelerated recovery from their desensitization, and augmented P2X3 receptor expression at membrane level due to facilitated trafficking of such molecules via intracellular activation of PKA and PKC dependent processes [16]. It is interesting that the P2X3 potentiation persists for hours after washout of CGRP, and further involves an increased synthesis of P2X3 receptors plus facilitated release of BDNF that exerts its own algogenic effect [93]. These observations outline a basic cellular process for extended sensitization of trigeminal sensory neurons to subsequent noxious stimuli.

One further synergic purinergic mechanism presumably closely involved in the algogenic effect of CGRP is mediated by the metabotropic P2Y receptors of glial cells. In fact, the strong algogenic peptide bradykinin stimulates release of CGRP by neurons to engage P2Y receptors of satellite glial cells with subsequent increase in intracellular Ca^2+^ and liberation of inflammatory cytokines that can sustain and amplify pain mechanisms [95]. Interestingly, activation of P2X3 receptors in the trigeminal ganglion can also release CGRP and dilate the middle meningeal artery [96], thus prolonging local neuroinflammation and neuronal sensitization.

These observations based on in vitro experiments provide a set of molecular mechanisms to support the pivotal role of CGRP in migraine, suggest a close coupling among neuronal and glial processes, and are consistent with the present treatment of migraine with pharmacological blockers of CGRP [13, 97].

The scenario of pain inducing agents impacting on P2X receptors is broad and should include the neurotrophin NGF that, independent from CGRP activity, produces multiple effects on such receptors of trigeminal sensory neurons. Thus, NGF accelerates recovery from desensitization of P2X3 receptors and induces splice variants of the P2X2 subtype favouring the expression of heteromeric receptors [98]. Nevertheless, it is difficult to employ anti-NGF treatment for pain suppression as NGF produces many beneficial effects, in particular, via TrkA receptor activity. However, the latter limitation could be overcome by uncoupling TrkA from PLCγ signalling without inhibiting the TrkA catalytic activity [99]. This approach should avoid the unwanted effects of direct TrkA inhibition and probably can be used for anti-nociceptive applications in migraine and other pain conditions. It is noteworthy that NGF can be used without eliciting significant pain when it is administered as human NGF mutant (hNGF P61F) that has minimal nociceptive action, thus making it an interesting candidate for clinical use in NGF-deficit conditions without affecting the nociceptive system [100].

While endogenous substances like CGRP, BDNF or NGF might operate sequentially or in parallel to trigger trigeminal pain, an important downstream process accompanying their action is the generation of an inflammatory milieu contributing to the establishment and extension of nociceptive dysfunction. This concept accords with the original hypothesis that chronic migraine is caused by an ongoing “sterile inflammation” that renders patients susceptible to frequent relapse [101–103]. In support of this theory, adding inflammatory cells like macrophages to cultured trigeminal neurons enhances P2X3 receptor activity likely because of the inflammatory agents released by such cells [104]. Thus, application of a standard inflammatory agent like lipopolysaccharide to trigeminal neurons slowly evokes a rise in functional responses of P2X3 receptors [105] probably in view of the action of TNFα, released during inflammation to strongly sensitize trigeminal sensory neurons [105–108].

It should be noted that these data, indicating new mechanisms of purinergic modulation, were obtained on neurons isolated mainly from young animals and, therefore, need further validation in in vivo models of migraine, including adult animals.

### P2X3 receptors in familial type migraine

A relatively rare type of migraine is familial hemiplegic migraine (FHM), a severe, monogenic disease that comprises three subtypes among which type 1 (FHM1) is the most frequently observed [109]. A widely studied mutation found in FHM1 is the R192Q of the *Cacna1a* gene coding for the α1 subunit of Ca_v_2.1 channels [110] that confers a gain of function to these voltage gated channels predominantly expressed by neurons [111].

Generation of knockin (KI) mice postnatally expressing this mutation has provided a powerful model of migraine [109] as these animals present symptoms consistent with human migraine [112]. Furthermore, cultured trigeminal neurons from FHM1 mice show strong functional upregulation of their P2X3 receptors [113] likely due to enhanced basal levels of CGRP and BDNF [114] that is translated into increased excitability with stronger action potential firing of trigeminal ganglion neurons [115].

Nonetheless, testing CGRP release in FHM1 mouse tissues has revealed a complexity of this mechanisms likely related to the age of animals and methodological considerations that include the sampled area, the low yield of endogenous peptide and the origin of CGRP. Indeed, Fioretti et al. (2011) have reported no difference in basal CGRP release from WT or KI trigeminal ganglia although K^+^-evoked release was found to be larger from KI ganglia [116]. Conversely, Chan et al. (2019) have studied the central trigeminal nuclei in adult animals where they found no change in evoked CGRP release from KI tissue [117]. Perhaps the role of endogenous CGRP in this mouse model might be better clarified in the future by applying selective chemical antagonists of CGRP receptors to find out how neuronal responses are changed to indicate any constitutively higher or stimulus-dependent concentration of this neuropeptide.

The molecular mechanisms responsible for higher activity of P2X3 receptors in this transgenic model also include tighter association between P2X3 receptors and the calcium/calmodulin-dependent serine protein kinase (CASK) [118] that leads to preferential compartmentalization of P2X3 receptors to membrane lipid rafts and more efficient P2X3 receptor function [119].

The phenotype produced by the R192Q mutation is, however, complex because it includes not only factors upregulating P2X3 receptors but also mechanisms that dysregulate their constitutive inhibition. One of them is represented by the brain natriuretic peptide (BNP), a blood borne peptide whose membrane receptors are strongly co-expressed with P2X3 receptors of trigeminal sensory neurons [120]. Via cGMP-dependent intracellular pathways, BNP constitutively limits the activity of P2X3 receptors, a process that is largely depressed in the FHM1 phenotype [120]. In addition, FHM1 mice show upregulation of the Na_v_1.7 subtype of voltage gated sodium channel [121] that contributes to their enhanced excitability.

Thus, the FHM1 mouse model has allowed identification of a series of dysregulated molecular mechanisms that synergize to facilitate firing of trigeminal sensory neurons, and it has provided a useful tool to test novel therapeutic approaches. The FHM1 model is, therefore, an experimental channelopathy that can be most useful to understand the role of Ca_v_2.1 channels not only in migraine [122] but also in a variety of other neurological syndromes like some forms of epilepsy, ataxia and dystonia [123].

### Endogenous P2X3 antagonists and pharmacological perspectives in migraine

Transmembrane P2X3 receptors with their extensive extracellular domain [124] contains several sites for binding allosteric modulators. Of special interest to the aims of the current review are modulators which might be used to control migraine pain generation. Notably, given the specific properties of ionotropic P2X3 receptors (very fast desensitization with slow recovery), these agents, apart from classical competitive and non-competitive antagonism, might selectively modulate desensitization. For instance, inhibition of P2X3 receptors mediated signalling could either include promotion of desensitization onset or slowing down the recovery process (reviewed in [125]). Several studies suggested pharmacological and native substances to target desensitization in order to obtain an antinociceptive action [126, 127].

Magnesium and calcium ions are the potent modulators of P2X3 receptor function providing opposite effects on receptor recovery from desensitization [128, 129]. The magnesium effect is of special interest to migraine pathology as some studies have suggested that migraine is associated with magnesium deficiency [130]. However, there are contrasting views on the use of magnesium as an aid to preventive migraine therapy [131, 132]. Magnesium is the natural blocker of NMDA receptors, which are one of the main glutamate receptor subtypes in the CNS and the main determinants of CSD related to migraine aura [133, 134]. Our previous studies indicated that magnesium deprivation promotes glutamate induced firing of nerve terminals via NMDA receptors [135] suggesting its role in the control of excitability of meningeal afferents. Magnesium can, however, directly inhibit P2X3 receptors [129], making it difficult to select the preferred target of anti-nociception against a potential sensitization of trigeminal firing. In contrast, extracellular calcium can strongly accelerate recovery from desensitization of P2X3 receptors [128], apparently competing with magnesium [129]. An analysis of genetic co-heritability and causality using data from the International Headache Consortium (23,285 cases, 95,425 controls) and circulating serum calcium levels (39,400 subjects) has revealed co-occurrence of migraine and hypercalcaemia, and it has suggested a causal link and increased risk of migraine with high serum calcium [136]. Of interest is the ability of bone cancer treatment drugs bisphosphonates to promote synthesis of the ATP endogenous analogue ApppI, which potently and specifically inhibits P2X3 receptors of trigeminal neurons [127].

P2X3 receptors are highly sensitive to changes in ambient temperature. Early studies with mice lacking P2X3 receptors have indicated that these transgenic animals are unable to code warm stimuli [21]. Subsequent studies have identified molecular mechanisms underlying the unusual sensitivity of P2X3 to temperature. Thus, while the onset of desensitization appears to be apparently temperature insensitive, recovery from desensitization accelerates with heat (Q_10_ ~ 10) [137]. Another interesting observation is that HAD by ambient nanomolar ATP which limits the function of P2X3 receptors [50], is much less effective at normal body temperatures [137]. Using total internal reflection fluorescence microscopy coupled with functional recovery after photobleaching, we have found that the peri-membrane turnover of P2X3 receptors had Q_10_ ∼4.5 suggesting that P2X3 receptor trafficking to plasma membrane is also highly temperature-sensitive [138]. These data also suggest the potential role of P2X3 receptors in the well-known analgesic effects of cooling.

It is well stabilised that females are more prone to migraine than men [139, 140]. Menstrual migraine is a primary headache especially difficult to treat and often persistent [141]. Because the level of female hormones (progesterone and oestrogens) falls in the perimenstrual period [142], oestrogen replacement therapy has been suggested to inhibit migraine pain [141]. Accumulating data indicate that oestrogen regulates P2X receptors through genomic and non-genomic pathways [143, 144], potentially contributing to the sex difference in pain and probably in migraine susceptibility. However, to date, there is no systematic analysis of sexual dimorphism of P2X3 receptors in the trigeminal nociceptive system.

Since the discovery of the selective expression of P2X3 receptors in sensory neurons, many research groups have focused on the development of specific antagonists aiming to inhibit painful signalling [58, 145–150]. Potentially, these selective P2X3 and P2X2/3 inhibitors may have implication for the treatment of migraine pain. Nevertheless, to the best of our knowledge, no clinical trials have been reported testing P2X3 antagonists in migraine. At the present time, these studies mainly deal with treating chronic cough and hypertension. For instance, potent P2X3 and P2X2/3 inhibitors are already at an advanced stage of clinical trial for the treatment of chronic cough [151, 152]. The main side effect of P2X2/3 antagonists is the loss of taste sensation [151] although the selective P2X3 antagonist BLU-5937 is apparently lacking such an effect [153]. It is, however, clear that there is a dearth of studies with selective P2X3 receptor antagonists in migraine pain either experimentally or clinically. A void that the present review might help to fill.

## Conclusions

P2X3 receptors selectively expressed in sensory neurons and enriched in the trigeminal nociceptive system have unique functional characteristics including synergy with pronociceptive CGRP signalling. Thus, the P2X3 receptor represents an attractive molecular target for innovative approaches to inhibit trigeminal pain in migraine. Future studies in vivo should investigate the therapeutic potential of blocking selectively P2X3 or P2X2/3 receptors in migraine pathology to validate their potential translational value.

## Data Availability

Not applicable.

## Abbreviations

CGRP: Calcitonin gene related peptide
NTPDase: Ecto-nucleoside triphosphate diphosphohydrolase
cAMP: Cyclic adenosine monophosphate
HAD: High affinity desensitization
α,β-meATP: α,β-Methylene-adenosine 5'-triphosphate
BzATP: 2ʹ(3ʹ)-O-(4-Benzoylbenzoyl) adenosine-­5ʹ-triphosphate
TNFα: Tumor necrosis factor alpha
Il-10: Interleukin 10
CSD: Cortical spreading depression
PKA: Protein kinase A
PKC: Protein kinase C
BDNF: Brain-derived neurothrophic factor
NGF: Nerve growth factor
TrkA: Tropomyosin receptor kinase A
PLCγ: Phospholipase C gamma
FHM: Familial hemiplegic migraine
CASK: Calcium/calmodulin-dependent serine protein kinase
BNP: Brain natriuretic peptide
cGMP: Cyclic guanosine monophosphate
ApppI: 1-Adenosine-5′-yl ester 3-(3-methylbut-3-enyl) triphosphoric acid diester
